# Biomarkers and Diagnostic Testing for Renal Disease in Sjogren's Syndrome

**DOI:** 10.3389/fimmu.2020.562101

**Published:** 2020-09-17

**Authors:** Giacomo Ramponi, Marco Folci, Salvatore Badalamenti, Claudio Angelini, Enrico Brunetta

**Affiliations:** ^1^Department of Nephrology, Humanitas Clinical and Research Center - Istituto di Ricovero e Cura a Carattere Scientifico (IRCCS), Milan, Italy; ^2^Department of Biomedical Sciences, Humanitas University, Milan, Italy; ^3^Department of Internal Medicine and Hepatology, Humanitas Clinical and Research Center - IRCCS, Milan, Italy

**Keywords:** biomarkers, renal disease, diagnostic test, autoimmunity, Sjogren's syndrome

## Abstract

Primary Sjogren's syndrome (pSS) is an autoimmune disorder in which lymphocytic infiltration leads to lacrimal and salivary glands dysfunction, which results in symptoms of dryness (xerophthalmia and xerostomia). Extraglandular features are common and may affect several organs. Renal involvement has long been known as one of the systemic complications of pSS. The most classical lesion observed in pSS is tubulointerstitial nephritis (TIN) and less frequently membranoproliferative glomerulonephritis (MPGN), which is related to cryoglobulinemia. In some cases, renal biopsy is necessary for the definitive diagnosis of kidney involvement. Patients may present with proximal renal tubular acidosis, distal renal tubular acidosis and chronic kidney disease. Response to treatment is usually favorable. However, occasionally severe and rarely lethal outcomes have been described. Recently, several case series and cross-sectional studies have been published which investigated the factors associated with renal involvement in pSS and the most accurate screening tests for early detection. The presence of xerophthalmia, anti-SSA and rheumatoid factor positivity, low C3 levels and other features have all shown either positive or inverse associations with the development of renal complications. Serum creatinine, alpha-1-microglobulin, cystatin-C have been evaluated as early detection biomarkers with variable accuracy. More advanced techniques may be necessary to confirm proximal and distal renal tubular acidosis, along with nephrogenic diabetes insipidus. The aim of the current paper is to summarize and critically examine these findings in order to provide updated guidance on serum biomarkers and further testing for kidney involvement in pSS.

## Introduction

Sjögren's syndrome (SS) is a systemic autoimmune disease which primarily causes dysfunction of exocrine glands. This leads to dryness of the ocular and oral mucosa (xerophthalmia and xerostomia), along with possible involvement of the pharynx, larynx, and vagina (1).

Furthermore, SS can also be complicated by severe manifestations such as multiorgan involvement and hematologic malignancies (2). A number of genetic and environmental factors may intertwine in the etiology of SS. The disease predominantly affects females (in a 9/1 ratio) in their middle age, but it can also affect different populations (1).

When SS affects a previously healthy individual, it is defined primary SS (pSS). When it affects patients suffering from another connective tissue disease, such as systemic lupus erythematous (SLE) or rheumatoid arthritis (RA), it is considered to be secondary SS (sSS) (1).

The vast majority of patients present with a combination of xerostomia and xerophthalmia (sicca syndrome). These may include the sensation of sand or gravel in the eye or the need to drink liquids frequently to contrast oral dryness (1).

Systemic manifestations of SS include cryoglobulinemic purpura, non-erosive symmetrical arthritis, interstitial lung disease, Raynaud's phenomenon, pericarditis, autoimmune hepatitis and primary biliary cirrhosis, polyneuropathy, thyroiditis, B cell lymphoma, autoimmune haemolytic anemia, and eventually renal disease, ranging from glomerulonephritis to tubulointerstitial nephritis (1, 3, 4).

Renal involvement has long been known to be multifaceted and possibly underestimated in SS. This review aims to shed light on the most recent developments in the field, with a special focus on biomarkers and diagnostic testing necessary to elicit subclinical renal damage (5, 6).

## Renal Disease in Sjogren's Syndrome: Glomerular and Tubulointerstitial Involvement

**Figure 1:**
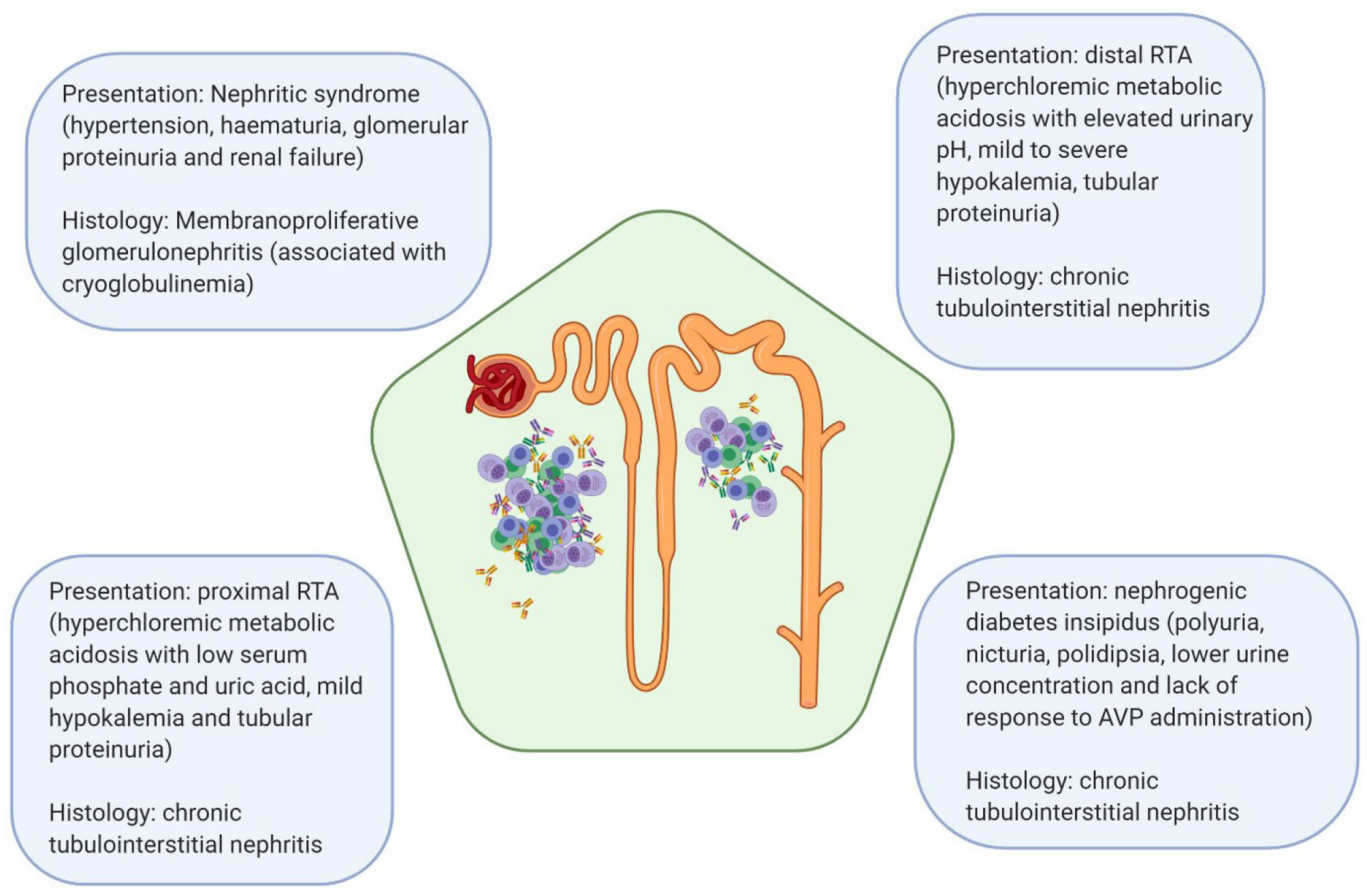
Most common clinical manifestations of renal disease in pSS and their associated pathologic lesions. RTA, renal tubular acidosis; AVP, arginine vasopressin.

A variety of renal manifestations has been described in pSS, with chronic and acute tubulointerstitial nephritis being the most common ones. Although less common, glomerular involvement has also been described, often in the setting of cryoglobulinemia (5–7). Most common presentations of kidney disease in pSS and their corresponding histological features have been depicted in Figure 1. The prevalence of renal involvement in pSS has been difficult to assess reliably, mostly due to changes in diagnostic criteria in the last 20 years and the presence of numerous studies with small number of patients or mixed cohorts of pSS and sSS (7).

According to some retrospective registries, kidneys would be affected in about 1% of pSS patients (8). However, according to several European studies, this may be as high as 5–14% (7, 9, 10). Interestingly, the prevalence of renal involvement in pSS was found to be more than 30% in a cohort of 573 Chinese patients (11). This variation in estimates may be attributed to use of different diagnostic criteria or underdiagnosis of subclinical tubular disease, which cannot be detected with standard kidney function screening tests (7). A different hypothesis, which is considered further in the article, is that ethnicity may play a role in the predisposition to renal disease in pSS (12). This would explain the wide variation in prevalence among studies performed in different world regions.

Symptomatic manifestations of renal dysfunction usually affect middle-aged patients, several years after the onset of pSS (7, 10, 13). These are included within the ESSDAI score, which tracks the activity of the disease. Renal activity of disease can range from absent to high, with low and moderate levels in-between. The considered factors are presence of haematuria and proteinuria (indicative of glomerular disease), renal failure, tubular acidosis (detected by hyperchloremic metabolic acidosis) and clearly histological evidence of active renal lesions (glomerulonephritis or interstitial lymphoid infiltrate) (9).

In histopathological studies, chronic tubulointerstitial nephritis was the most common finding (5, 13, 14). This is characterized by the presence of small lymphocytes (mixed T cells and B cells), plasma cells, and monocytes infiltrating the renal interstitium, together with atrophy of the tubules and fibrosis (5). These alterations imply a mixture of active inflammation and residual damage.

Tubulointerstitial nephritis usually presents insidiously, with tubular proteinuria (which cannot be readily identified by urine testing sticks due to the absence of albumin), renal tubular acidosis (RTA) and electrolytes disturbances (7, 15). A characteristic finding of TIN in pSS is the presence of distal RTA (dRTA, or type I RTA), which presents with an elevated urinary pH (>5.5) in the setting of an hyperchloremic metabolic acidosis (6, 16).

Unfortunately, the incomplete variant of dRTA may be more subtle and requires urinary acidification testing to be elicited (urinary pH persistently elevated after ammonium chloride administration) (6). Among these patients, hypokalemia due to dysfunction of the distal tubules H+/K+-ATPase is very common (almost 50%, according to one study) (13). When hypokalemia is associated with metabolic acidosis, an unusual finding, it is strongly suggestive of RTA.

Hypokalemia can also cause hypokalemic muscle paralysis, with generalized muscle weakness. Respiratory muscles can be affected as well. In one case, a patient died of cardiac arrest due to hypokalemia caused by dRTA in pSS (13). More commonly, dRTA can present with nephrolithiasis (7). This is caused by hypercalciuria and hypocitraturia. Consequently, renal stones in pSS may raise the suspicion of TIN.

Although less frequent, nephrogenic diabetes insipidus (nDI) and proximal RTA (pRTA, or type II RTA) can also be manifestations of chronic TIN in Sjogren's syndrome. In one study, testing with vasopressin unmasked a urinary concentration defect in ~20% of the participants (6). In a different cohort, nicturia, and lower urine concentration were observed in more than 80% of the patients (13). Proximal RTA is caused by ineffective bicarbonate resorption in the proximal tubule and can be distinguished from dRTA mostly due to low serum levels of phosphate and uric acid, which are similarly involved in its pathogenesis (7, 16).

Glomerular disease in pSS primarily takes the form of membranoproliferative glomerulonephritis (MPGN) and was observed in a substantial minority of patients in several bioptic studies (5, 8, 13, 14). MPGN is most commonly induced by cryoglobulinemia and manifests with features of nephritic syndrome (hypertension, haematuria, acute renal failure) or rarely rapidly progressive glomerulonephritis (when a rapid decline in glomerular filtration rate, GFR, is present) (7). In MPGN, the mesangial cells proliferate abnormally and the glomerulus is infiltrated with macrophages, with a consequent increase in the amount of mesangial matrix and a thickening of the basement membrane (5). In some cases, focal segmental glomerular sclerosis (FSGS) and membranous nephropathy (MN) have been observed in kidney biopsies of pSS patients (8, 14).

Patients who suffer from renal complications of Sjogren's disease are treated with corticosteroids, immunosuppressants, or a combination of both. Immunosuppressants described in literature include cyclophosphamide and, more recently, rituximab. Regardless of the treatment protocol and the underlying lesion, immunosuppressant treatment is uniformly regarded as effective and tolerably safe. According to researchers, most patients responded to treatment with an improvement in renal function and a reduction in proteinuria. Nevertheless, a small number of patients progressed to end-stage kidney disease despite immunosuppression (8, 13, 14).

## Testing For Renal Dysfunction in Sjogren's Syndrome: From Screening to Confirmation

In order to approach this topic, it is important to highlight how several disorders may mimic the systemic involvement and renal manifestations observed in pSS, which are not specific to it. Among these, the most important to keep in mind for the clinician are IgG4-associated disease, sarcoidosis, RA, and SLE (in which sSS may be present) (7). Therefore, diagnosis of pSS should be validated with the 2016 ACR/EULAR classification criteria [labial salivary gland biopsy with lymphocytic sialoadenitis (LSGB+), anti-SSA+, elevated ocular staining score, positive Schirmer test, and reduced salivary flow rate] (17).

Due to the progressive nature of renal disease in pSS and the overall excellent response to treatment, timely diagnosis is essential. It is suggested that screening for all patients should include yearly urinalysis and serum creatinine when manifestations of systemic disease are present (7, 8).

Furthermore, serum electrolytes should be measured in all patients at least yearly in order to detect disturbances due to TIN presenting as RTA. These should not be limited to measurements of sodium and potassium but should include chloride and bicarbonate. This will allow detection of hyperchloremic metabolic acidosis and potential hypokalemia (7). Although these recommendations are solely based on the opinion of experts opinions, with the lack of strong evidence supporting them, it is reasonable to aim for an early recognition of TIN, in order to administer steroids before irreversible fibrosis ensues.

In that subset of patients in whom abnormalities of renal function are detected, testing should be performed twice a year, with further examinations. Phosphate and uric acid, relevant to diagnosis of pRTA, should be included in the serum panel. Furthermore, urinary analysis of pH, osmolality, proteinuria, creatininuria, calciuria, citraturia, urinary sediment, and culture should be performed (7). As it was mentioned before, testing for proteinuria should not be specific for albumin, so that tubular proteinuria can be reliably diagnosed. Renal echography should be similarly performed twice a year if hypercalciuria is present, to rule out nephrolithiasis (7). In this case, a nephrologist may be consulted and kidney biopsy taken into consideration.

When hyperchloremic acidosis is detected in the patient's serum, the following diagnostic approach may be helpful in evaluating the etiology (16, 18, 19). Firstly, the serum anion gap (AG) should be calculated to confirm the presence of an hyperchloremic, or normal AG, metabolic acidosis. This is performed by subtracting Cl^−^ and ${\text{HCO}}_{3}^{-}$ to Na^+^ concentration. A normal AG is usually considered to be 8–12. Hypoalbuminemia may lead to pseudonormalization of the AG, so that 2.5 mEq/L should be added to the AG measurement for each 1 g/dL decrease in albumin levels from 4.5 g/dL.

The urine AG should then be measured (urinary Na^+^ + urinary K^+^ – urinary Cl^−^). When this is abnormally elevated, the suspicion of a dRTA ensues. On the contrary, if the urinary AG is normal, the cause of the acidosis is likely extrarenal (e.g., gastrointestinal fluid losses) or proximal tubular bicarbonate loss. Urinary pH should then be measured. Even though it is abnormally elevated in all forms of renal acidosis, it can rise above 5.3 only in distal RTA (19). If doubts persist, ammonium chloride, and IV sodium bicarbonate testing may be performed, although their execution and interpretation may require the aid of a nephrologist (19).

Hypophosphatemia can be observed in both dRTA and pRTA. Hypophosphatemia may lead to acquired hypophosphatemic osteomalacia, a disease of bone metabolism which presents with bone pain, weakness and increased susceptibility to fractures. In one review, 38 cases of pSS presenting with osteomalacia were reported (20). Most of these patients developed osteomalacia in the setting of dRTA, although few suffered from Fanconi's syndrome. In a different case report, a patient with pSS was reported to suffer from osteomalacia in the setting of Fanconi's syndrome, which led to hypophosphatemia and metabolic acidosis (21). Interestingly, hypocalcaemia was absent due to a secondary increase in parathyroid hormone levels.

## Predisposing Factors and Emerging Biomarkers

Recently, several original studies were published by Chinese researchers (Jing Luo and colleagues) which focused upon factors associated with renal disease and plausible biomarkers of subclinical renal inflammation in pSS (22–25).

In 2015, Zhao et al. evaluated in a cross-sectional study which factors were associated with systemic involvement in pSS (23). Although researchers did not focus specifically on renal disease, they found higher rates of systemic involvement in patients with anti-SSB and parotid enlargement or purpura at presentation. Interestingly, they evaluated the correlation of flaccid paralysis due to hypokalemia with the main immunological markers of pSS (anti-SSA, antinuclear antibodies, rheumatoid factor, low complement, and hypergammaglobulinemia). Due to the strict theoretical correlation of hypokalemic flaccid paralysis with dRTA due to chronic TIN, hypokalemic paralysis may be assumed to be a surrogate of renal damage. Flaccid paralysis was associated with anti-SSA positivity and rheumatoid factor (RF) positivity, while it did not show any association with antinuclear antibodies (ANA) and low complement (C3/C4) or hypergammaglobulinemia (hyperIgG) (23).

Three years later, a retrospective study conducted on 103 patients who had undergone kidney biopsy was published by Yang et al. (22). When patients with biopsy-proven renal disease were compared with a control group of pSS patients by means of univariate analysis (which may affect the reliability of results), several associations emerged. Patients with renal disease had significantly lower rates of interstitial lung disease and generally lung involvement, along with lower rates of leukopenia, objective xerostomia, xerophthalmia and hypergammaglobulinemia. In contrast, they had strikingly higher rates of corticosteroids treatment. Indeed, 96.1% of renal cases were treated with corticosteroids in comparison with 23.3% of non-renal cases (22). Clearly, it is difficult to draw any meaningful conclusion on pSS subsets when such an asymmetry in the studied populations, capable of explaining many of the previous differences, is present. Though, it is notable how renal involvement appears to be a strong driver of corticosteroids treatment in pSS, at least in this cohort.

A group of researchers led by Luo published two retrospective studies, in 2019, addressing the specific issue of renal involvement in pSS (24, 25). In one of these, 434 pSS patients (217 of which suffering from renal involvement) are compared in a multivariate analysis to detect clinical, serological and immunological factors associated with renal disease (25). Xerophthalmia and anti-SSA/Ro52 were found to be negatively associated with renal involvement while histological positivity of LSGB, reduced C3, hypoalbuminemia and anemia were all significantly more common in the renal disease group. Treatment with steroids or other immunosuppressants was not considered by the investigators.

In the other study, 1,002 patients were investigated with a similar approach (24). However, researchers extended the number of clinical features considered and also included possible biomarkers of early renal damage within the studied variables. Patients with renal disease were found to have higher serum levels of prealbumin, anti-scl-70, RF, anti-extractable nuclear antigen (ENA), anti-SSB, anti-SM, urea, creatinine, cystatin C, α_1_-microglobulin (α_1_-MG), serum β_2_-microglobulin, and other molecules. Reduced hemoglobin and C3 levels were also more common in the renal cases. On the contrary, anti-SSA were more common in patients without renal disease.

In order to evaluate the sensitivity of serum biomarkers for renal dysfunction in patients with known renal disease, receiver operating characteristic (ROC) curve was drawn for creatinine, cystatin C, α_1_-MG and various combinations of these. Area under the curve was best for the combination of creatinine and α_1_-MG, with a significant difference compared to creatinine alone (0.824 vs. 0.777) (24).

In 2018, a study was published in which B-cell activity markers and organ involvement in pSS were considered (26). Levels of serum free light chains (FLC) and β2-microglobulin (β2M) were found to be associated with renal disease. While renal impairment may itself lead to increased levels of FLC and β2M, authors corrected for glomerular function and results remained significant.

In 2019, another retrospective study was published which evaluated epidemiological factors such as age, ethnicity, gender, and latitude and their association with organ involvement in pSS (12). The study was based upon an existing registry of pSS patients (Sjogren Big Data Consortium) and included 10,007 patients from all over the world (although European patients were more represented than others). Renal involvement was significantly associated with younger age at diagnosis. Besides, individuals of Asian ethnicity were also at considerably higher risk of developing renal disease (10.2% compared with 3.8% in Whites, 2.2% in Hispanics and 1.4% in African Americans) (12).

For what concerns the latitude at which study participants lived, or North-South gradient, patients who lived in southern regions were significantly more affected by systemic involvement of pSS, including renal disease. Remarkably, this applied to Europe and Asia while it was not observed in America. This was the first time a North-South gradient of disease severity was observed in pSS.

Recently, the possible association of anti-SSA positivity with increased renal involvement was partially supported by a new experiment. Indeed, expression of two micro ribonucleic acids (miRNA), molecules involved in the regulation of gene expression, was found to be elevated in patients with pSS and anti-SSA (27). Patients with high expression of miR-146a and miR-4484 were found to have higher rates of renal disease and to be associated with anti-SSA positive pSS. Unfortunately, the control group was composed of patients not suffering from pSS and it is therefore difficult to assess the role of these molecules within the inflammatory process.

In a different study, it was noted that increased tubulointerstitial complement deposition (C4d in the absence of C1q) could be observed in most patients suffering from pSS renal disease (28). Investigators hypothesized a role for the mannose binding lectin pathway of complement activation. If this were confirmed by other studies, more extensive complement testing than C3 and C4 alone may become indicated in pSS. Relevant predisposition factors and exploratory biomarkers of kidney damage were summarized in Table 1.

**Table 1:** Summary of most relevant findings from recent studies on kidney disease in pSS.

**Author**	**Patient number**	**Country**	**Year**	**Factors positively associated with kidney disease**	**Factors inversely associated with kidney disease**	**Serum and urine biomarkers**
Zhao et al.	483	China	2015	Anti-SSA, RF		
Yang et al.	103	China	2018	Steroids treatment	ILD, xerostomia, xerophthalmia, hyperIgG	
James et al.	839	United Kingdom	2018			Serum free light chains, β2-microglobulin
Zeron et al.	10007	Worldwide (7,289 Europeans)	2019	Asian ethnicity, southern countries, young age at diagnosis	Whites, Hispanics and African Americans; Northern countries, older age at diagnosis	
Luo et al.	434	China	2019	LSGB+, low C3, hypoalbuminemia, anemia	Xerophthalmia, anti-SSA	
Luo et al.	1002	China	2019	Prealbumin, anti-scl-70, RF, ENA, anti-SSB, anti-SM, urea, creatinine, cystatin C, α_1_-MG, serum β_2_-microglobulin, anemia, low C3	Anti-SSA	Combination of serum creatinine and urine α_1_-MG

*Zhao, Yang, and Luo considered in their analyses the most common clinical and laboratory features which can be altered in pSS. James et al. considered markers of B-cell activation (BAFF, FLC, and β2M). Zeron et al. considered epidemiological factors and latitude in their study*.

## Discussion

It was long debated whether increased prevalence of kidney disease in Asian patients was due to methodological issues or a true difference in the underlying numbers. Apparently, Asian patients are indeed more at risk of renal disease, which seems to be one of the main pSS's complications in this subset of patients (7, 12). This may have some genetic reasons, although environmental factors are also conceivable (29). Along with Asian ethnicity, young age at diagnosis and residence in southern countries seem to be predisposing factors (12). While younger age at diagnosis may simply be the consequence of a more aggressive disease course, it is completely unclear why latitude is associated with systemic involvement.

Whether anti-SSA are associated with renal disease is still unknown. While two studies found a negative correlation of anti-SSA with renal disease, in one study a positive correlation was noted (with flaccid paralysis due to hypokalemia) (23–25).

In two studies, levels of C3 were found to be reduced more commonly in patients with renal disease than in the other group (24, 25). While this is contradicted by another study, it may further support the notion that complement activation plays a role in Sjogren's nephritis (23, 28). As complement pathways are multiple, it is unknown which of those is most involved and through which mechanisms.

A promising combination of biomarkers may be the utilization of combined serum creatinine and urinary α_1_-MG testing. Although the addition of urinary α_1_-MG would increase the cost of testing, it would allow to detect early tubular dysfunction with a better accuracy than creatinine alone (24). As α_1_-MG is not an acute phase protein, variations in its urinary levels can be safely inferred not to be caused by a transient state of systemic inflammation (30). While FLC and β2M testing may also be associated with renal damage, their role as diagnostic biomarkers was not considered yet (26).

Limitations of these discoveries should be considered. While one study was probably flawed by the huge difference in corticosteroids treatment between the patients and control group, it is even more remarkable that this issue was not considered in the following ones (23–25). Steroids act on most organ systems with deep immune and hematological implications. The same applies to other immunosuppressants. It would therefore be fundamental to consider the impact of treatment on proposed associations.

Furthermore, little is known about the relevance of biomarkers and predisposing factors in predicting the response to treatment and the overall outcome. Creatinine levels and proteinuria, when present, are known to improve with treatment of the underlying renal inflammation, as it may be expected (8, 13, 14). However, no study has been yet performed with the aim of quantifying how these molecules levels (e.g., creatinine, α_1_-MG, FLC, β2M) may affect treatment outcome or prognosis.

Another issue is the low quality of the studies reviewed, which are mostly cross-sectional retrospective studies. This study design does not allow to draw meaningful cause-effect relationships and is also subject to all the possible bias of retrospective studies. Eventually, in one study it was proposed that patients with anti-dsDNA in pSS are at higher risk of developing renal disease. Due to the strong association of anti-dsDNA with SLE, it is plausible that these patients suffered from sSS in the setting of SLE (31).

All in all, it is evident that an increase in interest for pSS-associated renal disease took place in the last few years. This was mostly driven by Chinese researchers, possibly because of the higher incidence of the disease in their population. Although findings may still be difficult to implement in clinical practice, new and intriguing possibilities emerged with these which certainly deserve further investigations.

## Author Contributions

GR, MF, and CA contributed substantially to the conception and design of the review. EB and SB supervised and provided critical revision of the article. GR and MF wrote the manuscript with support from EB. All authors contributed to the article and approved the submitted version.

## Conflict of Interest

The authors declare that the research was conducted in the absence of any commercial or financial relationships that could be construed as a potential conflict of interest.
